# Effective Therapeutic Strategies to Prevent Frailty and Falls in Community-Dwelling Older Adults

**DOI:** 10.14336/AD.2025.0445

**Published:** 2026-05-12

**Authors:** Dmitry Tsvetkov, Marleen J. Meyer-Tönnies, Mladen V. Tzvetkov, Werner Weitschies, Stefan Engeli, Anna Lebedeva, Anke Hannemann, Martin C. Jordan, Ulrike Garscha, Luzia Valentini, Daria Antonenko, Lieven N. Kennes, Maik Gollasch

**Affiliations:** ^1^Department of Internal Medicine and Geriatrics, University Medicine Greifswald, Germany; ^2^Department of General Pharmacology, Institute of Pharmacology, University Medicine Greifswald, Germany; ^3^Department of Biopharmaceutics and Pharmaceutical Technology, Institute of Pharmacy, University Medicine Greifswald, Germany; ^4^Department of Clinical Pharmacology, Institute of Pharmacology, University Medicine Greifswald, Germany; ^5^Department of Internal Medicine B, Cardiology, University Medicine Greifswald, Germany; ^6^Institute of Clinical Chemistry and Laboratory Medicine, University Medicine Greifswald, Greifswald, Germany; ^7^DZHK (German Centre for Cardiovascular Research), partner site Greifswald, Greifswald, Germany; ^8^Department of Traumatology, University Medicine Greifswald, Greifswald, Germany; ^9^Department of Pharmaceutical/Medicinal Chemistry, Institute of Pharmacy, Greifswald University, Greifswald, Germany; ^10^Department of Agriculture and Food Sciences, Neubrandenburg Institute of Evidence-Based Nutrition (NIED), University of Applied Sciences Neubrandenburg, Neubrandenburg, Germany; ^11^Department of Neurology, University Medicine Greifswald, Germany; ^12^Department of Economics and Business Administration, University of Applied Sciences Stralsund, Stralsund, Germany

**Keywords:** frailty, falls, kidney function, medication, polypharmacy, potentially inappropriate medication, orthostatic hypotension, physical exercise, osteoporosis, fractures, malnutrition, cognitive training, brain stimulation, machine learning, periadventitial dysfunction, heart failure with preserved ejection fraction, HFpEF

## Abstract

Frailty and the consequent risk of falls represent significant challenges for community-dwelling older adults, often leading to severe injuries, functional decline, and loss of independence. Falls typically result from multiple interacting risk factors, many of which are modifiable through targeted interventions. This mini-review focuses on evidence from randomized controlled trials evaluating effective therapeutic strategies to prevent frailty and falls. Comprehensive assessment and management of modifiable risk factors have been shown to significantly reduce fall incidence. Key interventions include community-based and home-based exercise programs emphasizing balance and strength training. Additionally, the treatment of osteoporosis is crucial to reducing the risk of fall-related fractures. Other modifiable risk factors, such as orthostatic hypotension, polypharmacy, environmental hazards, osteoporosis, malnutrition, and cognitive impairment, require targeted assessment and intervention. Despite these advances, further research is needed to optimize multifactorial interventions and tailor strategies to individual risk profiles. Innovative research directions now span from micro to macro levels, incorporating insights from animal models to human studies, aiming to unravel underlying mechanisms and develop personalized therapeutic strategies. This review discusses emerging evidence and new interdisciplinary research avenues that offer hope for mitigating frailty and preventing falls in community-dwelling older adults.

## 1. Introduction

Frailty is recognized as a clinical state preceding disability [1-3], though both conditions may coexist [4, 5]. It is a dynamic continuum ranging from robustness to severe frailty, with the potential for reversibility [4]. Addressing frailty early is critical, as functional decline associated with frailty is a major contributor to disability and fall risk [6]. The two most widely used frailty assessment tools in research and clinical practice are the physical frailty phenotype [2] and the Frailty Index (FI) of accumulated deficits [7, 8].

Frailty is an age-related syndrome marked by diminished physiological reserves across multiple organ systems, leading to increased vulnerability to stressors [4, 9]. Preventing or slowing its progression before substantial functional decline occurs is a key priority in healthcare. Falls are a major consequence of frailty, affecting one third of community-dwelling adults aged 65 and older annually, with 10% experiencing multiple falls [10]. Consequences of falls range from activity restriction and medical attention to severe injuries such as fractures [11]. In some cases, prolonged immobility post-fall can lead to complications like rhabdomyolysis and renal failure [12]. Fear of falling, which develops in 21–39% of fallers, further exacerbates physical decline and reduces quality of life [13].

**Figure 1.**
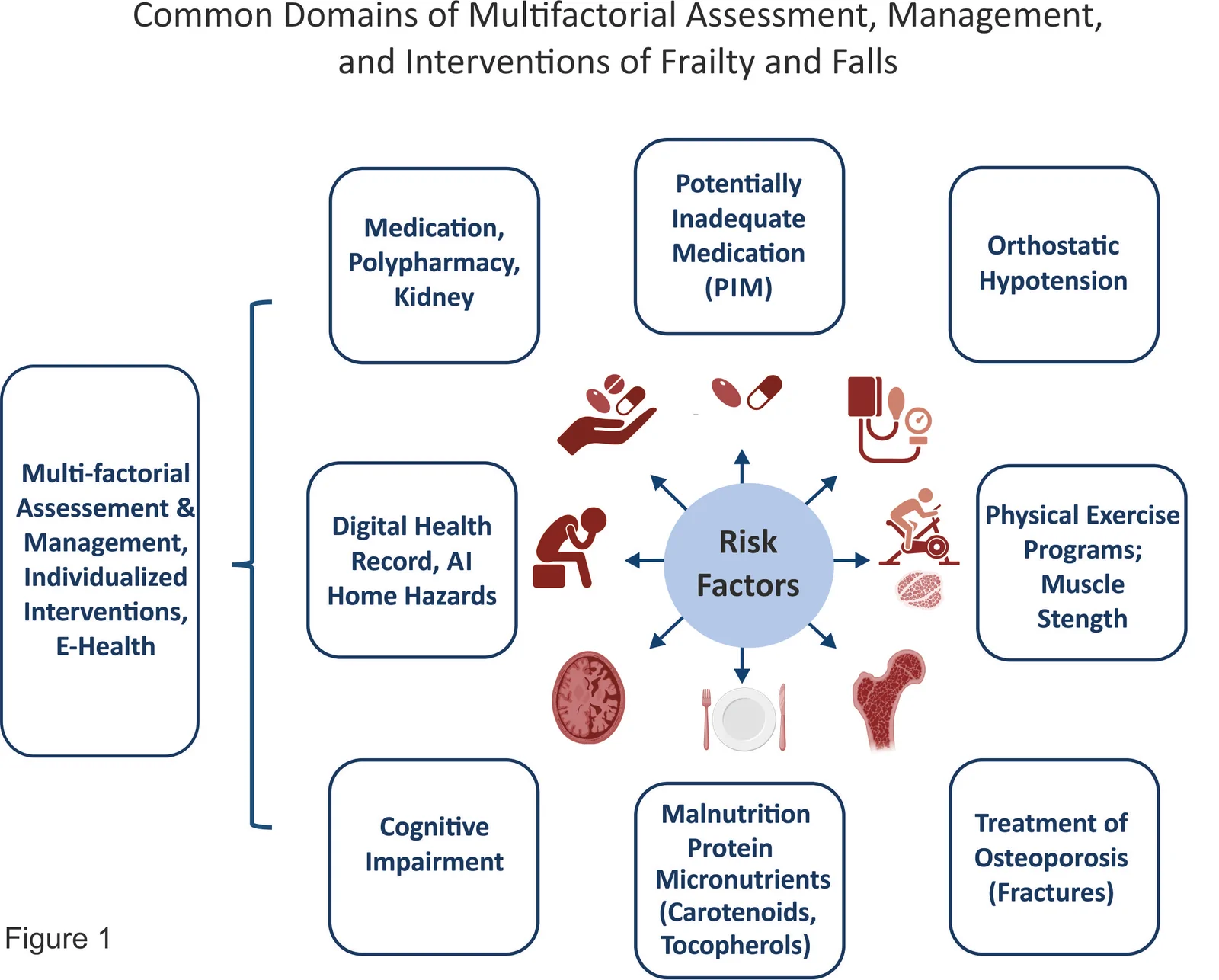
**Common domains of multifactorial assessment, management, and interventions.** This figure illustrates the primary risk factors for falls that are most frequently assessed in clinical and research settings, as reviewed in this paper based on evidence from randomized controlled trials. It provides an overview of targeted interventions and their impact on fall prevention. Given the time-intensive nature of comprehensive multifactorial assessment and management, a modular approach—distributing evaluations across multiple clinical visits—may improve feasibility and adherence. Furthermore, advancements in technology offer novel opportunities for intervention implementation, while ongoing research continues to refine our understanding of the mechanisms driving intervention effectiveness, ultimately guiding the development of more effective strategies for preventing frailty and falls in older adults. Intrinsic risk factors encompass age-related changes such as balance impairment, muscle weakness, orthostatic hypotension, cognitive decline, and other components of frailty that contribute to diminished resilience. Extrinsic risk factors, on the other hand, include environmental hazards, which further elevate the risk of falling. Given the multifactorial nature of falls, a strategic focus on modifiable factors that represent the final common pathways to falls is crucial. These key risk factors are also the primary targets in randomized controlled trials evaluating fall and frailty prevention interventions.

Most falls arise from a combination of intrinsic (e.g., gait and balance impairment) and extrinsic (e.g., environmental hazards) factors (Fig. 1) [14]. Multifactorial risk assessment highlights a core set of modifiable risk factors frequently addressed in randomized trials, including medication effects, alcohol consumption, visual deficits, cognitive impairments, malnutrition and cardiovascular conditions [14, 15]. Osteoporosis, a significant factor in fall-related fractures, becomes increasingly relevant with aging, thereby elevating the risk profile (Fig. 1).

While some frailty interventions remain unproven, consensus guidelines suggest integrating feasible, cost-effective strategies into clinical practice [4]. It is crucial to ensure that frailty does not serve as a barrier to appropriate interventions, reinforcing ageism in healthcare [4, 16]. Future research must refine targeted prevention strategies, spanning micro- to macro-level approaches—from mechanistic studies in animal models to clinical trials in human populations. This review explores emerging interdisciplinary research directions that hold promise for mitigating frailty and preventing falls in community-dwelling older adults.

## 2. Multifactorial Assessment and Management in Fall Prevention: Evidence-Based Approaches

Early prevention strategies are crucial in mitigating fall risks among older adults. In clinical practice, a comprehensive assessment is crucial for identifying individuals at high risk of falls. Simple screening questions, evaluation of predisposing factors (e.g., medication use, alcohol consumption), and precipitating factors (e.g., preceding symptoms, environmental hazards) provide essential insights into fall risk. Circumstances surrounding the fall, presence of associated injuries or loss of consciousness, along with a physical examination and office-based tests of gait, balance, and strength, are routinely indicated for patients with a history of falls or fear of falling that limits daily activities. These factors serve as markers of heightened risk, warranting multifactorial intervention.

Multifactorial Assessment and Management of key fall risk factors, followed by targeted Interventions based on identified risks, is recommended for high-risk individuals [17]. A meta-analysis of 19 trials demonstrated that multifactorial assessment and management significantly reduced fall rates compared to usual care or interventions not specifically designed to prevent falls [18]. In addition to focusing on the most commonly assessed risk factors, as recently summarized by Ganz et al. [17], this review further expands and refines these perspectives. These key domains, illustrated in Figure 1, represent evidence-based strategies designed to mitigate fall risk and enhance functional outcomes. Clinical trials have consistently shown that targeted interventions addressing these domains are more effective than usual care in preventing falls and improving overall mobility in older adults. The effective strategies include medication review and deprescribing, management of polypharmacy and potentially inappropriate medications, treatment of orthostatic hypotension, structured physical exercise programs, osteoporosis and fracture prevention, nutritional optimization, and cognitive training. Particular attention is required for older adults with reduced kidney function, which affects drug elimination and is common in old adults [19]. Additionally, HFpEF (Heart Failure with Preserved Ejection Fraction), is a prevalent and underdiagnosed condition with a poor prognosis [20]. HFpEF is associated with upstream mediators of inflammation and exercise intolerance, which may contribute to increased fall risk (Fig. 1) [21].

These key domains provide a foundation for addressing evidence–practice gaps and guiding innovative research directions. Future investigations should integrate micro- to macro-level approaches, leveraging insights from animal models to human clinical trials to unravel underlying mechanisms and develop personalized preventive and therapeutic strategies. This review explores emerging interdisciplinary research avenues that offer new hope for mitigating frailty and preventing falls in community-dwelling older adults.

### 2.1 Medication, Polypharmacy and Kidney function

Medication use, particularly polypharmacy (Fig. 1), is a significant contributor to falling risk in older adults [22, 23]. A structured medication review is essential to identify medications without a clear indication, optimize therapy, and minimize adverse effects. Age-related changes in drug pharmacokinetics are well recognized [24]. However, patients aged 75 and older are significantly underrepresented in clinical trials during drug development [25], making evidence-based dose adjustments in this population challenging [26]. Kidney function plays a crucial role in drug clearance, rendering careful dose adjustments necessary to avoid drug accumulation and toxicity. Polypharmacy is an issue for all patients with chronic kidney disease (CKD) [27]. It is associated with worse clinical outcomes and lower quality of life in kidney and old patients [27]. Polypharmacy is also linked to an increased risk of kidney failure, cardiovascular events, and all-cause mortality in patients with non-dialysis-dependent CKD [28, 29]. Close monitoring of renal function, individualized medication adjustments, and patient-centered deprescribing strategies are essential components of a multifactorial fall prevention approach. The cited trials and evidence present both strengths and limitations. A strength of the cited research is the clear identification of polypharmacy as a major risk factor for falls and adverse outcomes in older adults. However, limitations emerge from the heavy reliance on observational studies, which restricts the ability to establish definitive causal relationships. Additionally, the persistent underrepresentation of adults over 75 in clinical trials creates critical gaps in our understanding of medication safety, efficacy, and appropriate dosing in this vulnerable population.

Two key research directions for optimizing medication use, particularly polypharmacy, are understanding the (i) aged kidney and (ii) the role of pharmacogenetics and changes in pharmacokinetics in older adults. Investigating age-related changes in renal function is crucial, as declining kidney function affects drug clearance, increasing the risk of accumulation and toxicity. Additionally, pharmacogenetic variations influence drug metabolism, efficacy, and adverse reactions, necessitating a more personalized approach to prescribing in older adults. These research areas are essential for improving medication safety, reducing adverse drug reactions (ADRs), and enhancing therapeutic outcomes in aging populations.

#### Aged kidney:

Age-related CKD is characterized by persistent renal inflammation and tubulointerstitial fibrosis, driven by a complex interplay of myofibroblasts, immune cells, and endothelial cells, resulting in excess extracellular matrix (ECM) deposition and kidney dysfunction [30]. The exact origin of ECM-producing cells remains controversial, with contributions from fibroblasts, pericytes, mesenchymal stem cells (MSCs), and perhaps endothelial-to-mesenchymal transition (EndMT) processes [31, 32]. Recent evidence highlights Transient Receptor Potential Cation Channel 6 (TRPC6)[33] as a novel key player in fibrogenesis, with TRPC6 inhibition protecting against fibrosis in murine hearts and kidneys [34-36]. We examined TRPC6 in acute kidney injury [37] and renal fibrosis [34]. TRPC6 blockade reduces fibrosis by ~30% [34, 36]. However, the precise renoprotective mechanisms remain unclear. Future research should focus on elucidating the role of TRPC6 in kidney endothelial cells during fibrotic remodeling, integrating endothelial dysfunction into kidney fibrosis pathways to identify novel therapeutic targets for age-related CKD.

#### Pharmacogenetics and changes in pharmacokinetics in older adults

Multiple studies over the past 20 years have shown that pharmacogenetic (PGx) testing can reduce drug toxicity and improve efficacy [38, 39] [40]. A recent randomized controlled trial demonstrated that considering PGx markers during prescribing reduced clinically relevant ADRs by 30% [41]. However, the effects of PGx in older adults remain debated. While theoretically declining kidney and liver function in older adults was thought to overrule PGx effects, recent studies showed that PGx effects in older adults may be similar or even stronger. In patients over 75 years, CYP2C19 polymorphisms were linked to a higher risk of major adverse cardiac events under clopidogrel after percutaneous coronary intervention (PCI) [42]. However, age-stratified analyses of pharmacogenetic randomized controlled trials (RCTs) suggested that different dose-tailoring algorithms may be needed for the older adults [43]. Although meta-analyses on PGx and aging exist [44], direct comparative studies are still missing. We previously showed that PGx markers in drug-metabolizing enzymes and transporters significantly affect drug pharmacokinetics and pharmacodynamics in young healthy volunteers [45-48]. However, the extent of these effects in older adults remains unclear. In rats, we found age- and sex-related differences in pharmaco-kinetics, partially explained by variations in mRNA expression [49]. In a population-based cohort of older dementia patients (DelpHi-MV study), we found that 6% of participants carried a high-risk actionable PGx marker while taking a drug known to be affected by this genotype [50]. These individuals could potentially benefit from PGx-guided prescribing. Future studies should investigate the combined effects of aging and pharmacogenetics on drug pharmacokinetics, with a particular focus on whether high-risk PGx genotypes contribute to an increased risk of ADRs in older adults with polypharmacy, falls, and frailty. Classical pharmacokinetic studies can be challenging in older adults due to the higher number of concomitant medications. However, microdosing approaches offer a promising alternative [51]. The use of biomarkers may also represent a feasible strategy for optimizing drug dosing in older adults [52]. Understanding these interactions will be crucial for optimizing personalized medication management and improving drug safety in the aging population.

### 2.2 Potentially Inappropriate Medication

Potentially inappropriate medication (PIM) is a concept to identify drugs which pose a potentially high risk of ADRs in older patients. Several research groups and national societies have developed PIM lists. These lists are typically developed through a consensus process including a limited number of experts and undergo regular updating. Unfortunately, when different PIM lists are compared, the overlap varies largely (reviewed in [53]). Despite variations in definitions and classifications, the use of potentially inappropriate medication remains prevalent, as illustrated in Figure 1. [54] The prevalence of PIM use varies significantly across countries, with estimates ranging from 9% in Germany to 81% in Australia [55]. Drugs related to the central nervous system and cardiovascular disease, benzodiazepines, analgesics, and nonsteroidal anti-inflammatory drugs are the most commonly used PIMs [17, 55]. A key intervention is weighing the risks and benefits of these medications, with an emphasis on dose reduction or discontinuation when possible. The evidence-based approach is supported by guidelines, e.g. the Deprescribing Network and Bruyère Research Institute's deprescribing guidelines [56-58]. The evidence from the cited trials includes both robust findings and recognized limitations. One randomized controlled trial (RCT) evaluating psychotropic medication withdrawal found that participants in the withdrawal group had a lower fall rate compared to those who continued their medication. However, 47% of participants resumed psychotropic medications within a month after the trial concluded, despite the initial fall reduction benefits [59]. This underscores the challenges of deprescribing, particularly when these medications are used for managing chronic symptoms [60]. A recent Cochrane review on the evidence from interventions targeting the appropriate use of polypharmacy for older people did not show significant improvements through interventions in most questions covered. However, the main message of this review was that evidence quality is largely low to very low based on the analyzed study characteristics [61]. Whereas a gradual tapering approach, patient education, and alternative non-pharmacologic interventions may enhance the success of medication withdrawal while minimizing withdrawal effects and symptom recurrence, this multifactorial approach still needs to be thoroughly tested in high quality RCTs. Efforts to reduce potentially inappropriate medications (PIMs) in older adults face challenges. Current barriers include insufficient knowledge and training in deprescribing, time constraints, communication gaps, fears of negative outcomes, and resistance from patients [62]. Variations in PIM lists and study designs, along with small samples and limited blinding, reduce the reliability of findings and highlight the need for coordinated, high-quality research to improve prescribing practices.

Medication management in older adults is increasingly complex due to polypharmacy, drug-drug interactions, and PIM prescriptions [63, 64]. Poor adherence and medication errors arise from cognitive decline, multiple prescribers, and frequent generic substitutions, leading to ADR and reduced treatment efficacy [65]. While these issues are well described qualitatively, there is a lack of interventional research to improve medication safety and optimize pharmacotherapy. Future studies should aim to investigate the fundamental mechanisms underlying ADR, optimize medication use, and improve pharmacotherapy in older patients on all relevant levels. Our previous research has focused on drug absorption under real-life conditions in older patients, particularly within the European Training Network AGePOP [66] [67]. Analyzing age-related factors affecting medication intake and formulation preferences [68-70] are also important to develop medications accepted by older individuals. Developing methodologies for assessing gastric emptying [71, 72], conducting medication-use surveys, and analyzing changes in PIM prescriptions over time using data from the Studying Health in Pomerania (SHIP) study, a population-based cohort in our area [73] represent important tesserae to advance medication safety in older individuals.

Another critical area of investigation involves the objective assessment of suspected ADR in cohort participants and geriatric patients, providing real-world data on the clinical impact of medication-related complications. That approach should be supplemented by well attended and documented n-of-1 trials to evaluate the efficacy of deprescribing interventions, allowing for personalized pharmacotherapy adjustments that optimize treatment while minimizing harm.

### 2.3 Orthostatic Hypotension

Orthostatic hypotension is characterized by a persistent decrease in systolic blood pressure of 20 mm Hg or more or diastolic blood pressure of at least 10 mm Hg within three minutes of standing. Some individuals may experience an initial drop in blood pressure upon standing that resolves within three minutes; in such cases, patients should be counseled to stand up gradually and delay walking immediately to minimize dizziness and the risk of falling[17, 74-76]. For patients with confirmed orthostatic hypotension (Fig. 1), a careful medication review is critical. Drugs that may contribute to the condition, especially those with anticholinergic effects or strong antagonism at alpha1-adrenergic receptors, should be discontinued whenever possible [77]. Additionally, water drinking is a fundamental preventive measure [78]. If symptoms persist despite these interventions or if patients exhibit significant blood pressure fluctuations, such as transitioning from high blood pressure while lying down to hypotension when standing, further diagnostic evaluation is necessary. Referral for assessment of neurogenic causes may be appropriate, and in severe or treatment-resistant cases, pharmacologic therapy should be considered [79, 80]. A multidisciplinary approach, combining lifestyle modifications, medication adjustments, and personalized management strategies, is essential for effectively addressing orthostatic hypotension and preventing associated complications [17, 79]. There are notable strengths and limitations within the cited studies and supporting evidence. A key strength is the depth of physiological and clinical characterization in many of the trials, often involving thorough assessments and deep phenotyping, which enhances the understanding of orthostatic hypotension mechanisms and management. However, these studies often include a relatively small number of patients or participants, limiting statistical power and the generalizability of findings to broader clinical populations. Additionally, variability in diagnostic criteria and outcome measures across studies can hinder comparisons and the development of standardized treatment protocols. The pathophysiology of orthostatic hypotension is multifaceted, involving impairments in autonomic regulation, vascular responsiveness, and fluid balance. Recent studies indicate that increased arterial stiffness significantly contributes to its development, with findings showing a 40% higher risk of orthostatic hypotension in adults with elevated arterial stiffness [81], highlighting the critical role of vascular integrity in blood pressure stabilization.

Beyond arterial stiffness, perivascular adipose tissue (PVAT) is increasingly recognized as a key regulator of vascular homeostasis. PVAT exerts its effects by releasing bioactive molecules that influence smooth muscle contractility, helping to maintain arterial tone and vascular responsiveness [82]. However, conditions such as aging and chronic kidney disease (CKD) lead to PVAT dysfunction, which is characterized by increased inflammation, oxidative stress, and enhanced secretion of vasoconstrictive adipokines [83, 84]. These changes promote vascular stiffness and arterial tone dysregulation [82]. PVAT dysfunction exacerbates vascular aging, potentially exacerbating orthostatic hypotension and vascular aging [85, 86]. Aging is also associated with altered arterial calcium signaling [87], which affects vascular function and may influence orthostatic blood pressure regulation. In chronic kidney disease (CKD), shifts in oxylipin profiles—bioactive lipid mediators that regulate vascular tone—further contribute to vascular dysfunction and increased cardiovascular risk [88]. Studies have demonstrated that PVAT loses its protective anticontractile properties in these conditions, leading to heightened vasoconstriction and impaired blood flow regulation [85, 86].

Our previous research identified that PVAT releases perivascular relaxing factors (PVRFs) that activate potassium (KCNQ5) channels in vascular smooth muscle cells, promoting vasodilation [82, 89, 90]. These findings suggest that PVAT dysfunction, arterial stiffness, and PVRFs (oxylipin) imbalances collectively contribute to vascular dysregulation [83, 91], increasing the likelihood of orthostatic hypotension in older and CKD populations. To advance the understanding of paracrine dysregulation of PVAT in aging, we propose that a focused investigation into its role in vascular homeostasis is crucial. Investigating these pathways may uncover novel targets for therapeutic intervention. In addition, PVAT dysfunction and its contribution to arterial health and vascular aging should be assessed by integrating findings from animal models and human cohort studies. This approach would allow for the translation of experimental data into clinically relevant strategies to mitigate age-related vascular decline in orthostatic hypotension.

From our perspective, dysfunctional PVAT is an underappreciated yet significant contributor to vascular stiffness, arterial tone dysregulation, and impaired blood pressure homeostasis, particularly in aging and chronic kidney disease (CKD). We believe that targeting PVAT-signaling represents a promising therapeutic strategy to counteract vascular and metabolic disturbances. By restoring PVAT function, it may be possible to enhance vascular elasticity, stabilize blood pressure regulation, and reduce the risk of orthostatic hypotension, ultimately improving cardiovascular health and resilience in aging populations.

### 2.4 Physical Exercise and Cardiac Aging

A comprehensive fall-risk assessment should include brief screening tools and functional mobility tests, such as observing the patient rise from a chair, walk, and stand in progressively challenging positions (side-by-side, semitandem, and full tandem). Systematic reviews and meta-analyses of large randomized controlled trials (RCTs) consistently demonstrate the benefits of exercise - including group or home-based programs, outpatient or home physical therapy, and assistive devices - in prevention of both falls and cardiovascular disease (Fig. 1) [92, 93]. A meta-analysis of 59 RCTs confirmed that exercise reduces falls in individuals at average or high risk [94]. Evidence also suggests that exercise lowers fall-related fracture rates by 27% (based on 10 trials) and reduces medically attended falls by 39% (5 trials).[94] Both home-based programs (e.g., the Otago Exercise Program, Go4Life) and group-based programs have been shown to effectively reduce fall rates [92]. The most effective programs target balance and lower limb strength, incorporating progressively challenging exercises [92]. Tai Chi has been associated with a 20% reduction in fall rates, as demonstrated across up to 24 clinical trials [92, 95]. However, walking alone has not been shown to prevent falls [17, 94]. There is little evidence supporting dance and yoga as an effective alternative to strength and balance training for fall prevention [96-98]. The cited research demonstrates a range of strengths as well as certain limitations. Many of the randomized controlled trials included in meta-analyses feature robust participant numbers and well-structured interventions, lending credibility to the observed benefits of exercise-based fall prevention. However, a common limitation is the relatively short duration of most interventions—typically only 3 to 4 months—leaving uncertainty about the long-term sustainability and effectiveness of these programs. Furthermore, while short-term gains in balance and strength are well documented, it remains unclear whether extending the duration or intensity of training leads to greater or more lasting reductions in fall risk. Assessing gait, balance, and strength can help determine whether patients can safely participate in unsupervised programs or require physical therapy supervision [99]. Patients without significant functional impairments may be appropriate candidates for home- or community-based fall prevention programs [17, 100, 101].

The American Heart Association recommends at least 150 minutes of moderate-intensity aerobic activity per week, such as 30 minutes a day, five times a week, to promote cardiovascular health. However, personalized exercise approaches are increasingly recognized for optimizing outcomes [102]. A major challenge arises in patients with heart failure with preserved ejection fraction (HFpEF) [20] - a common, underdiagnosed condition in older adults - which is characterized by exercise intolerance and poor prognosis [103, 104]. This limits the effectiveness of standard exercise programs in a population that could otherwise benefit greatly.

Current diagnostic approaches of HFpEF rely on resting morphological and hemodynamic parameters [105], yet many symptoms, particularly exercise intolerance, become apparent only under stress conditions [106-108]. Moreover, specific pathogenetic factors impede exercise tolerance in HFpEF, such as coronary microvascular dysfunction (CMD), a key contributor to HFpEF due to vascular aging, hormonal deficiency, proteostatic decline and inflammatory dysregulation [21, 109, 110]. The simultaneous assessment of myocardial exercise tolerance and biomarkers of microvascular changes using non-invasive vascular function tests [111-113] in HFpEF patients could enable early diagnosis and guide personalized interventions [21, 114], including structured exercise training [115], to improve vascular function and HFpEF prognosis. Our group has investigated non-invasive vascular reactivity techniques such as flow-mediated dilation of the brachial artery, reactive hyperemia with peripheral artery tonometry, and retinal dynamic vessel analysis, as clinically relevant in vascular ageing and cardiovascular diseases [116, 117]. We also showed that in HFpEF cohorts, exercise training resulted in distinct metabolic protein alterations [118] with high variability, potentially explained by differences in vascular dysfunction [115]. Future research should refine diagnostic and therapeutic strategies for HFpEF, particularly in vascular dysfunction and exercise intolerance [119, 120]. Assessing hemodynamic parameters and coronary microvascular function using non-invasive MRI-based techniques may enhance early detection and targeted interventions. Investigating peripheral microvascular function and serum biomarkers linked to vascular aging and HFpEF could further clarify disease mechanisms and identify novel diagnostic markers. Given that exercise intolerance limits frailty and fall prevention strategies, evaluating exercise tolerance in HFpEF patients with and without controlled physical training will determine the impact of tailored exercise programs. Although exercise has shown positive effects on cardiac aging, existing studies are far from perfect. Confounding factors - such as the absence of a standardized metric for healthy aging and the underrepresentation of populations from low- and middle-income countries - limit the generalizability of the findings [121]. There is growing recognition that individual responses to exercise are not uniform [122]. Genetic differences, other confounding factors significantly influence cardiovascular aging and it’s response to physical activity. However, these genetic variances are not yet routinely accounted for in clinical studies. This state-of-affairs limits our understanding of personalized effects. Fortunately, decreasing sequencing costs and advances in data collection, sharing, and analysis offer promising opportunities to better understand not only the mechanisms of healthy aging but also individual responses to interventions [123].

Additionally, developing a risk score for early HFpEF diagnosis and personalized exercise prescriptions by integrating hemodynamic, microvascular, and biomarker data may improve early intervention and individualized rehabilitation strategies. These efforts could enhance risk stratification, functional capacity, and cardiovascular resilience in older adults.

### 2.5 Osteoporosis & Fracture Treatment

Osteoporosis, characterized by bone deterioration, is a primary contributor to fragility fractures in older adults (Fig. 1). These fractures often lead to chronic pain, reduced mobility, increased mortality, and diminished quality of life [124]. In older adults, low body weight often relates to low muscle mass and function and poor physical performance, indicative for sarcopenia. Co-occurring with osteoporosis, this condition further increases fracture risk [125]. A body mass index (BMI) of 15 kg/m², compared to a BMI of 25 kg/m², is associated with a hazard ratio for hip fracture of 2.9 in women [126]. Our previous research has demonstrated that lower anthropometric measurements, including body mass index (BMI), are associated with a reduced bone stiffness index as measured by quantitative ultrasound at the heel [127]. Maintaining a body mass index (BMI) above 20 kg/m², yet below the obesity threshold, is recommended to minimize health risks [128]. Investigating the complex relationships among obesity, frailty, and osteoporosis is essential, as obesity can influence both bone health and physical function, thereby affecting frailty progression (Fig. 1). When adipogenesis outweighs osteoblasto-genesis, bone formation may decrease [129], resulting in impaired bone turnover. Clinical studies have demonstrated significant associations between pro-inflammatory adipokines and adverse bone outcomes, including reduced bone mineral density and increased fracture risk [130, 131]. Excessive lipid accumulation leading to bone marrow adiposity negatively impacts bone tissue [132]. Statins may enhance bone mineral density (BMD) and reduce fracture risk [129]. Obesity is linked to decreased vitamin D levels, crucial for calcium homeostasis and bone health [133].

#### Vitamin D

While earlier studies suggested that vitamin D supplementation reduces fall risk compared to control interventions, recent systematic reviews of randomized controlled trials (RCTs) challenge this notion. A comprehensive meta-analysis assessing vitamin D (and its analogues) for fall prevention in community-dwelling older adults without specific indications for supplementation found no consistent benefit [134, 135]. Among the trials analyzed, five showed no reduction in fall risk, one reported a decrease, and another observed an increase in falls [134, 135]. There are notable strengths and limitations within the cited studies and supporting evidence. A strength of the available research lies in the inclusion of large, well-conducted randomized controlled trials, which enhance the reliability of findings. However, significant heterogeneity exists across studies in terms of baseline vitamin D status, dosing regimens, duration of supplementation, and participant characteristics—all of which contribute to inconsistent outcomes. Additionally, many trials were not specifically designed to assess falls as a primary endpoint, limiting the interpretability of their findings for fall prevention. These variations make it difficult to draw definitive conclusions regarding the role of vitamin D supplementation in reducing fall risk across diverse older adult populations. Given this lack of clear efficacy, routine vitamin D supplementation solely for fall prevention and primary prevention of fractures (postmenopausal women) in community-dwelling older adults is not recommended [134-136]. However, emerging evidence from the DO-HEALTH Randomized Clinical Trial suggests that a combination of vitamin D3, marine omega-3 fatty acids, and a structured home exercise program (SHEP) may offer benefits in prevention of both frailty and falls among generally healthy older adults [137, 138]. All three treatments had additive benefits on measures of biological aging (PhenoAge, GrimAge, GrimAge2 and DunedinPACE) [139]. These findings highlight the potential for multimodal interventions in promoting musculoskeletal health and resilience in aging populations, warranting further investigation into synergistic effects beyond fall prevention alone.

#### Injury Prevention

Effective injury prevention in older adults should prioritize the assessment and management of fracture risk, particularly in those with a history of fragility fractures. Individuals with a previous vertebral or hip fracture resulting from minimal trauma should receive pharmacologic treatment for osteoporosis to reduce the likelihood of subsequent fractures. In the United States, bone mineral density (BMD) assessment is recommended for women aged 65 years and older or for those with major risk factors for osteoporosis, even in the absence of prior fractures, to facilitate early diagnosis and intervention [140, 141]. A meta-analysis of randomized controlled trials found no significant reduction in hip fracture risk with use of hip protectors [142]. Osteoporosis management, fall prevention strategies, and multimodal interventions remain the cornerstone of fracture risk reduction in aging individuals. In October 2023, the German-speaking scientific osteological societies (DVO) released an updated guideline on osteoporosis prevention, diagnosis, and treatment in postmenopausal women and men over 50 years. Key updates include a shift from 10-year to 3-year fracture risk assessment and revised treatment thresholds. For a 3-year fracture risk >10%, osteoanabolic therapy is recommended first, followed by antiresorptive therapy [128]. Pelvic and acetabular fractures in older adults are rising entities and pose challenges due to complex injuries, poor bone quality, and disability risks [143, 144]. No consensus exists on optimal treatment - conservative management, fixation, or joint replacement. Each approach has benefits and risks [145, 146]. The lack of clear evidence necessitates biomechanical, clinical, and registry-based research to investigate treatment mechanisms and optimize outcomes.

Future research should focus on optimizing fracture management in older patients through evidence-based treatment strategies. In biomechanical laboratories, different approaches using synthetic and cadaveric hip specimens can be applied to investigate trauma mechanisms, surgical care pathways, bone metabolism, and fixation failure. Key areas of study include metabolic age scores [147] and novel biomarkers [148, 149] to improve risk assessment. By integrating Omics, scRNA sequencing, biomarkers, imaging, and epidemiological analyses, causal mechanisms and clinically relevant risk factors can be identified, ultimately advancing prevention and treatment strategies.

### 2.6 Malnutrition & Nutrition Strategies

Dietary intake and quality are key contributors to frailty and fall risk in older adults (Fig. 1) [150]. Protein intake, a crucial component of nutritional guidelines for malnutrition and frailty, plays a central role in maintaining muscle mass and function [151]. Protein consumption below 1.2 g/kg/day has been significantly associated with increased frailty risk [152], highlighting the importance of adequate dietary protein. The Mediterranean diet, widely recognized as one of the healthiest dietary patterns, has shown promising benefits for frailty prevention [153, 154] [155]. A recent study demonstrated that adherence to the Mediterranean diet is associated with lower odds of frailty in older adults [156]. These findings suggest that nutritional strategies emphasizing protein intake and Mediterranean diet adherence may play a vital role in mitigating frailty and its associated complications. The DO-HEALTH Trial suggests that combining vitamin D3, omega-3 fatty acids, and a structured home exercise program (SHEP) may help prevent frailty and falls in generally healthy older adults [137, 138]. Given that protein sources vary in their beneficial and harmful components, maintaining dietary protein diversity may serve as a protective strategy against frailty [152].

Poor micronutrient status has been associated with frailty (Fig. 1) [157]. A recent study by Kuczmarski et al. found that an anti-inflammatory diet, rich in dietary antioxidants, fiber, vegetables, and fruits while low in ultra-processed foods, significantly reduced the risk of prefrailty and frailty in the longitudinal Healthy Aging in Neighborhoods of Diversity across the Life Span (HANDLS) study over an 8.7-year follow-up in middle-aged adults [158]. These findings align with data showing higher intake of antioxidants, including vitamins A, C, E, selenium, zinc, and carotenoids, have a significantly lower risk of developing frailty symptoms [159]. A review of 16 RCTs suggested that the higher the intake of omega-3 polyunsaturated fatty acids (PUFAs), the greater the improvement in upper-extremity muscle strength and lower-extremity physical function [160]. The intake of seafood/fish (with increased circulating levels of omega-3 PUFAs) is associated with lower measures of frailty [161, 162]. From a dietary perspective, current research indicates that frailty prevention and management should focus on adequate protein intake from diverse sources, including animal proteins (with limited red meat) and plant-based proteins. Additionally, a diet rich in anti-inflammatory and antioxidant nutrients, emphasizing fruits, vegetables, and healthy vegetable oils (e.g., the Mediterranean diet), is recommended to support muscle health and overall resilience in aging individuals [152].

The mechanisms of health effects of omega-3 and omega-6 fatty acids have been the subject of ongoing scientific debate for years [163]. While metabolites derived from arachidonic acid (omega 6-fatty acids), such as leukotrienes and prostaglandins, have traditionally been associated with pro-inflammatory processes [164], oxylipins derived from eicosapentaenoic acid (EPA) and docosahexaenoic acid (DHA), both omega 3-fatty acids, have been described to exert anti-inflammatory and health-promoting effects [165]. However, beyond these well-characterized C20- and C22-fatty acids, the biological relevance of C18-fatty acids, including linoleic acid (LA, omega 6) and α-linolenic acid (ALA, omega 3), and their metabolites, known as C18-oxylipins (octadecanoids), have remained largely underexplored. Emerging evidence suggests that octadecanoids influence inflammation, metabolism, cell proliferation, and pain modulation [166-171]. Additionally, LA has been linked to benefits in metabolic disorders, cardiovascular diseases, and cancer [172-174]. These findings challenge the oversimplified distinction of omega 3-fatty acids as beneficial and omega 6-fatty acids as harmful, highlighting the need for a more nuanced understanding of their physiological roles, particularly in the context of octadecanoids and their integration into healthy dietary patterns. Previous research investigated biological targets in fatty acid metabolism, explored the role of oxylipins in inflammation and developed pharmacological modulators using advanced techniques such as LC-MS/MS and SFC-MS analysis [175-180]. The impact of targeted nutritional strategies on inflamma-aging [181, 182] and malnutrition-sarcopenia has been investigated [183-185]. Future research could explore the role of octadecanoids in the health of older individuals by comparing their metabolic impact in those with and without age-related inflammation. This investigation may help clarify the mechanisms underlying the observed association between high adherence to the Mediterranean and similar dietary patterns and a significantly lower incidence of frailty in community-dwelling older adults. Furthermore, these insights could contribute to the development of innovative nutritional and therapeutic strategies aimed at modulating inflammation in aging organs, ultimately improving health outcomes in older adults.

Additionally, pharmacological interventions, including well-known inhibitors of the eicosanoid pathways such as soluble epoxide hydrolase inhibitors, cycloxygenase inhibitors and lipoxygenase inhibitors, should be utilized to distinguish enzymatic biosynthetic pathways from non-enzymatic oxylipin formation, aiming to identify novel drug targets. Translational research can be further advanced through cohort studies, with a particular emphasis on the interplay between Mediterranean dietary patterns, oxylipins and frailty, thereby bridging fundamental mechanistic insights with clinical applications.

Together, these approaches can contribute to the development of anti-inflammatory therapies and the identification of mechanisms that counteract age-related organ and tissue deterioration, ultimately promoting healthier aging.

### 2.6 Cognitive Training & Brain Stimulation

Cognitive impairment is an independent risk factor for falls, regardless of the medications used to treat them (Fig. 1) [186, 187]. Brief screening tools, such as the Mini-Mental State Examination (MMSE), clock-drawing test, Mini-Cog for cognitive impairment provide efficient initial assessments in clinical practice [188]. Patients who screen positive should undergo further evaluation, including an assessment for reversible causes such as hypothyroidism or vitamin deficiencies, which can contribute to cognitive and mood disturbances. Given the association of antidepressants with increased fall risk [189] and cholinesterase inhibitors with a higher incidence of syncope [190], a nonpharmacologic approach should be prioritized when managing depression and cognitive decline [17]. Strategies for cognitive enhancement with potentially severe side effects (e.g., anti-amyloid treatments in patients with subjective or mild cognitive impairment or dementia) are often not justifiable in terms of risk-benefit ratio, and non-drug behavioral strategies, such as cognitive training are desirable. Cognitive enhancement interventions offer a means to improve memory functions, with significant implications for daily functioning [191, 192].

When combined with non-invasive brain stimulation, these interventions may yield longer-lasting effects that extend beyond the trained tasks to enhance other, "non-trained" cognitive abilities [193]. Notably, transcranial electrical stimulation (tES), a leading non-invasive brain stimulation technique, has shown promising results in augmenting cognitive performance in older adults, particularly when integrated with targeted training programs [194, 195]. On a cellular level, tES increases cortical excitability by changing membrane potentials toward depolarization, tuning ongoing neural processes, and promoting long-term-potentiation-like synaptic plasticity [196].

The cited research highlights a range of strengths alongside certain limitations. While the findings are promising, training programs are often demanding and prolonged, with inconclusive evidence regarding the sustainability and transferability of their effects [191]. Additionally, limitations exist in the methods used to define cognitive impairment, as well as in how fall outcomes are classified—both of which significantly influence risk quantification. Although strong evidence links global cognitive measures with serious fall-related injuries, there remains no consensus on specific threshold values [187].

The neural changes associated with aging result in distinct mechanisms underlying interventional effects, differing not only between older and younger brains but also among older individuals themselves [194, 197]. Understanding these mechanisms is crucial for the development of effective interventions. Additionally, growing evidence suggests that a one-size-fits-all approach may be suboptimal; instead, individualized protocols are increasingly recognized as essential to achieving maximal benefits [198]. However, the optimal stimulation parameters remain undefined, and studies employing prospectively personalized protocols have yet to be conducted. The clinical relevance of these interventions is constrained by their ability to produce lasting effects, a challenge that remains under active investigation. To date, cognitive improvements resulting from intensive training protocols combined with brain stimulation over several weeks typically persist for only 1-3 months [199, 200]. Incorporating “booster” training sessions—repeating the intervention at scheduled intervals—holds potential for inducing more durable, long-term effects [201]. However, this hypothesis warrants exploration in future research.

Together, tES-assisted cognitive interventions have the potential to improve cognitive functioning beyond the trained task and thus reduce frailty in community-dwelling older adults.

## 3. Biostatistics & Machine Learning

For all evidence-based approaches above, biostatistics plays a pivotal role. The discrimination of treatment effects from random noise, the identification of patterns within complex datasets and ultimately any sophisticated evidence-based decision-making relies on solid, unbiased data analyses. While biostatistics, in its broad and general sense, remains a cornerstone of medical research, artificial intelligence (AI) has advanced rapidly in recent years, emerging as a transformative force in many areas including medicine. Here, a key strength of future collaborative projects will emerge. With the extensive information and data collected from the different risk and research domains above, novel approaches will be able to apply a powerful unsupervised machine learning (ML) algorithm to generate two distinct clusters, aiming to distinguish frail vs non-frail patients. Unlike conventional classifications such as Fried et al. [2] or Rockwood et al. [7, 8] this AI-driven approach is entirely data-driven and objective, relying on evidence rather than predefined criteria (Fig. 1). Thus, instead of advancing isolated projects independently, integrating them into a cohesive framework unlocks unparalleled synergies for this ML-procedure, maximizing insights and efficiency.

Furthermore, for supervised ML-procedures, especially in the data-sensitive field of medicine, comprehensibility of and trust in AI-results are imperative, while powerful techniques like neural networks and deep learning become increasingly intransparent. Explainable AI (XAI) bridges theses technological advancements with aspects of human perception and interaction. [202] Techniques such as feature attribution methods (e.g., LIME, SHAP), neural saliency mapping, and rule-based approaches have been developed to provide insights into the inner workings of models. Collaboration of clinicians with statisticians should assess the practical comprehensibility of different XAI techniques. These efforts will enhance AI useability, making it safer and more interpretable for clinical trials and medical decision-making. Explanations however vary due to factors like data dependency, model architecture, explanation methods, and stochastic influences. Future research should also aim to analyze and minimize these variations using statistical techniques, improving the reliability and stability of XAI systems.

## 4. Digital Health in the Prevention of Falls and Frailty

Digital health technologies are revolutionizing the prevention of falls and frailty, particularly in rural areas, where access to specialized healthcare is often limited. Wearable sensors, mobile health applications, and remote monitoring systems enable continuous assessment of mobility, balance, and physical activity, allowing for early detection of functional decline and timely, personalized interventions [203-206]. Motion-sensing technologies, such as accelerometers and gyroscopes in smart devices, can track gait patterns and postural stability, identifying subtle changes that indicate increased fall risk [207]. These insights facilitate real-time feedback and adaptive interventions [208], including tailored physical exercise programs [209] that enhance strength, balance, and mobility, helping older adults maintain independence and reduce the likelihood of institutionalization.

Televisits and telemedicine expand access to fall prevention programs, including virtual physiotherapy, cognitive training, and medication reviews, ensuring that older adults—especially those in rural areas—receive timely and evidence-based care [210-212]. AI-driven fall risk prediction models integrate data from wearables, electronic health records, and patient-reported outcomes, enabling individualized exercise prescriptions and long-term monitoring of adherence [213, 214]. Supervised home-based exercise interventions, such as Otago or structured balance training, can be delivered *via* digital platforms, improving long-term engagement and effectiveness [215].

In addition to mobility support, digital health addresses vision and hearing impairments, which are key yet often overlooked contributors to fall risk. Virtual screenings and Artificial (AI)-enhanced assistive devices, such as smart hearing aids and visual aids, help mitigate sensory deficits [216, 217]. Home modification occupational therapy counseling is essential in adapting living environments for safety, integrating fall detection systems, automated lighting, and voice-activated assistants to support independent living [218].

By combining telemedicine, digital monitoring, tailored digital physical exercise interventions, sensory support, and home modifications, digital health offers a comprehensive, data-driven approach to reducing frailty and falls. These innovations enable older adults to live safely and independently in their own homes for longer, delaying or avoiding the need for nursing home placement, and ultimately improving mobility, accessibility, and quality of life in aging populations, particularly in underserved rural communities.

## 5. Concluding remarks

This mini-review has underscored the critical domains in the prevention of falls and frailty in older adults, emphasizing the importance of multifactorial interventions over standard care. Effective strategies include medication review and deprescribing, polypharmacy management, treatment of orthostatic hypotension, structured physical exercise, osteoporosis and fracture prevention, nutritional optimization, and cognitive training. Special consideration is needed for individuals with reduced kidney function, which significantly influences drug metabolism and overall treatment efficacy. Furthermore, HFpEF, an underdiagnosed condition with poor prognosis, emerges as a crucial factor, given its association with inflammation, exercise intolerance, and increased fall risk. These insights underscore the urgent need for a translational research approach to bridge evidence-practice gaps and refine personalized interventions. Future investigations should integrate micro- to macro-level methodologies, utilizing animal models and human clinical trials to uncover underlying mechanisms and develop targeted therapeutic strategies. By advancing precision medicine in geriatric care, these efforts hold the potential to enhance functional independence and overall health outcomes in aging populations.
